# Editorial: Molecular mechanisms of ion channel activation and modulation

**DOI:** 10.3389/fphys.2025.1695777

**Published:** 2025-09-19

**Authors:** Meng Cui, Miao Zhang

**Affiliations:** ^1^ Department of Pharmaceutical Sciences and the Center for Drug Discovery, School of Pharmacy and Pharmaceutical Sciences, Bouvé College of Health Sciences, Northeastern University, Boston, MA, United States; ^2^ Department of Biomedical and Pharmaceutical Sciences, Chapman University School of Pharmacy, Irvine, CA, United States

**Keywords:** ion channel physiology, channel activation mechanisms, modulation of ion channels, redox signaling, lipid regulation, neuronal excitability

## Introduction

Ion channels are integral membrane proteins that regulate the passage of ions across cell membranes, thereby shaping processes as diverse as neuronal signaling, muscle contraction, and hormone secretion. Channel activation is typically initiated by a stimulus such as voltage, ligand binding, or mechanical stress that induces conformational changes and opens the channel pore. Beyond activation, channels are subject to fine-tuned modulation by a wide range of endogenous and exogenous agents, from lipids and neurotransmitters to pharmacological drugs. Modulators can stabilize the open, closed, or inactivated states, shift ligand affinities, or alter gating kinetics. Dissecting the structural and dynamic basis of these processes remains central to understanding ion channel physiology and to exploiting channels as therapeutic targets.

This Research Topic brings together studies that illuminate distinct aspects of ion channel activation and modulation, with a particular focus on ligand- and lipid-mediated regulation, redox modulation, and macromolecular channel complexes.

### Redox modulation of ion channels

Orfali et al. provide a comprehensive overview of how oxidative stress modulates ion channels in neurodegenerative diseases. Reactive oxygen and nitrogen species can act as modulators by covalently modifying amino acid residues, shifting channel gating, or disrupting ligand sensitivity. These insights highlight that oxidative stress is not merely a secondary consequence of pathology but a direct molecular mechanism influencing ion channel activation and dysfunction.

### Intracellular Ca^2+^ and neuronal excitability

Bertagna et al. apply the loose-patch clamp technique to probe hippocampal neurons, showing how intracellular Ca^2+^ release from ryanodine receptors contributes to modulation of voltage-gated Na^+^ and K^+^ currents. Here, Ca^2+^ acts as an intracellular ligand-like modulator, linking intracellular store signaling to channel activity and shaping neuronal excitability. This work exemplifies how intracellular signaling cascades serve as indirect modulators of ion channel activation.

### Macromolecular channel complexes

Stary-Weinzinger explores the structural basis of Nav1.5–KIR2.1 interactions, demonstrating how disease-associated mutations may impair complex formation and thereby modulate channel function. This perspective reframes ion channel modulation not only as a ligand- or lipid-driven process but also as one mediated by protein–protein interactions within higher-order channel complexes.

### Lipid regulation of GIRK2 channels

Cui et al. provide mechanistic insights into how cholesteryl hemisuccinate (CHS) enhances GIRK2 activation by stabilizing PIP_2_ binding and lowering the barrier for K^+^ permeation. Lipid modulators, by altering the conformational energy landscape of channels, represent an underexplored but powerful class of regulators. Using Molecular Dynamics (MD) simulations, this study directly connects structural binding events with dynamic conformational changes underlying channel activation.

### Connexin hemichannels in systemic disease

Balboa et al. highlight how connexin hemichannels contribute to early muscle dysfunction in sepsis. Their findings position hemichannels as modulatory conduits of inflammatory signaling, where pathological activation alters membrane permeability and triggers downstream atrophic pathways. This underscores the relevance of channel modulation in systemic disease contexts beyond excitable tissues.

## Conclusion

Together, these studies advance our understanding of the molecular mechanisms of ion channel activation and modulation across diverse contexts: redox signaling, intracellular Ca^2+^ release, protein–protein interactions, lipid binding, and systemic inflammation. The integration of structural, computational, and functional approaches across these works demonstrates how channel gating and modulation are tightly interwoven processes, shaped by both intrinsic channel architecture and extrinsic modulators. By deepening mechanistic insights, this Research Topic contributes directly to the broader goal of structure-based drug discovery and the development of novel therapeutic interventions targeting ion channels in human disease.

